# Kernel Probabilistic K-Means Clustering

**DOI:** 10.3390/s21051892

**Published:** 2021-03-08

**Authors:** Bowen Liu, Ting Zhang, Yujian Li, Zhaoying Liu, Zhilin Zhang

**Affiliations:** 1 Faculty of Information Technology, Beijing University of Technology, Beijing 100124, China; liubw2017@emails.bjut.edu.cn (B.L.); zhangting@bjut.edu.cn (T.Z.); zhaoying.liu@bjut.edu.cn (Z.L.); zhangzl@emails.bjut.edu.cn (Z.Z.); 2 School of Artificial Intelligence, Guilin University of Electronic Technology, Guilin 541004, China

**Keywords:** fuzzy c-means, kernel probabilistic k-means, nonlinear programming, fast active gradient projection

## Abstract

Kernel fuzzy c-means (KFCM) is a significantly improved version of fuzzy c-means (FCM) for processing linearly inseparable datasets. However, for fuzzification parameter m=1, the problem of KFCM (kernel fuzzy c-means) cannot be solved by Lagrangian optimization. To solve this problem, an equivalent model, called kernel probabilistic k-means (KPKM), is proposed here. The novel model relates KFCM to kernel k-means (KKM) in a unified mathematic framework. Moreover, the proposed KPKM can be addressed by the active gradient projection (AGP) method, which is a nonlinear programming technique with constraints of linear equalities and linear inequalities. To accelerate the AGP method, a fast AGP (FAGP) algorithm was designed. The proposed FAGP uses a maximum-step strategy to estimate the step length, and uses an iterative method to update the projection matrix. Experiments demonstrated the effectiveness of the proposed method through a performance comparison of KPKM with KFCM, KKM, FCM and k-means. Experiments showed that the proposed KPKM is able to find nonlinearly separable structures in synthetic datasets. Ten real UCI datasets were used in this study, and KPKM had better clustering performance on at least six datsets. The proposed fast AGP requires less running time than the original AGP, and it reduced running time by 76–95% on real datasets.

## 1. Introduction

Clustering is an important unsupervised method, and the purpose of clustering is to divide a dataset into multiple clusters (or classes) with high intra-cluster similarity and low inter-cluster similarity. There have been many clustering algorithms, such as k-means (KM) and its variants [1,2,3,4,5,6,7,8,9,10,11,12,13,14,15,16]. Others are based on minimal spanning trees [17,18,19], density analysis [20,21,22,23,24,25], spectral analysis [26,27], subspace clustering [28,29], etc.

Generally, k-means minimizes the sum of squared Euclidean distances between each sample point and its nearest clustering center [1]. One variant of k-means is kernel k-means (KKM) [30,31,32,33], which is able to find nonlinearly separable structures by using the kernel function method. Another variant of k-means is fuzzy c-means (FCM) [2], which determines partitions by computing the membership degree of each data point to each cluster. The higher the membership degree, the greater the possibility of the data point belonging to the cluster. Although FCM is more flexible in applications [11,12,13,14,15,16], it is primarily suitable for linearly separable datasets. Kernel fuzzy c-means (KFCM) [34] is a significantly improved version of fuzzy c-means for clustering linearly inseparable datasets. However, the problem of KFCM with fuzzification parameter m=1 cannot be solved by existing methods.

To solve the special case of KFCM for m=1, a novel model called kernel probabilistic k-means (KPKM) is proposed. In fact, KPKM is a nonlinear programming model constrained on linear equalities and linear inequalities, and it is equivalent to KFCM at *m* = 1. In theory, the proposed KPKM can be solved by the active gradient projection (AGP) method [35,36]. Since the AGP method may take too much time on large datasets, we further propose a fast AGP (FAGP) algorithm to solve KPKM more efficiently. Moreover, we report experiments demonstrating its effectiveness compared with KFCM, KKM, FCM, and KM.

The paper is organized as follows: Section 2 reviews previous work. The KPKM algorithm is proposed in Section 3. Section 4 proposes a solution for KPKM. Section 5 presents descriptions and analyses of experiments. Conclusions and future work are mentioned in Section 6.

## 2. Background and Related Work

There has been a lot of work related to this paper, mainly including k-means, fuzzy c-means, kernel k-means and kernel fuzzy c-means.

K-means minimizes the sum of squared Euclidean distances between each sample point and its nearest cluster center. K-means first chooses initial clustering centers randomly or manually, and then partitions a dataset into several clusters (a data point belongs to the cluster whose clustering center is nearest to the data point), and computes the mean of a cluster as the clustering center. K-means repeatedly updates clustering centers and clusters until convergence. FCM has the same ideal as k-means. FCM introduces a membership degree $w_{ij}$ and a fuzzy coefficient m into the objective function. The higher $w_{ij}$ is, the greater possibility of the *i*-th data point belonging to the *j*-th cluster. K-means and FCM belong to partition-based clustering algorithms, and partition-based clustering algorithms usually are not able to cluster linearly inseparable datasets. Kernel method maps a linearly inseparable dataset into a linearly separable space, so kernel k-means (and FCM) using a kernel function can cluster linearly inseparable datasets.

### 2.1. K-Means and Fuzzy C-Means

Let $X=\left\{x_{i}|x_{i}\in R^{D},1\leq i\leq L\right\}$ represent a dataset. K-means divides it into k clusters. If $\omega_{j}$ denotes the *j*-th cluster, we have $X=\overset{K}{\underset{j=1}{⋃}}\omega_{j}$ and $\;\forall 1\leq i\neq j\leq K,\omega_{i}⋂\omega_{j}=\emptyset$. Using $L_{j}=\left|\omega_{j}\right|$ to stand for the number of elements in $\omega_{j}$ with the center of $c_{j}$, k-means can be described as minimizing the following objective function:

(1) $$J=\sum_{i=1}^{K}\sum_{x_{i}\in \omega_{j}}{∥x_{i}-c_{j}∥}^{2}$$

where

(2) $$c_{j}=\frac{1}{L_{j}}\sum_{x_{i}\in \omega_{j}}x_{i}.$$

Let $L=\sum_{j=1}^{K}L_{j}$. By using c instead of k and assigning membership degree $w_{ij}$ to the *i*-th data point in the *j*-th cluster for 1≤i≤L and 1≤j≤C, the fuzzy c-means clustering model can be formulated as follows:

(3) $$\begin{array}{c} \min\;J=\sum_{j=1}^{C}\sum_{i=1}^{L}w_{{}_{ij}}^{m}{∥x_{i}-c_{j}∥}^{2}\\ s.t.\sum_{j=1}^{C}w_{ij}=1,w_{ij}\geq 0 \end{array}$$

where m>1, $w_{ij}$ and $c_{j}$ are computed alternately below [2].

(4) $$\left\{\begin{array}{c} w_{ij}=\frac{{∥x_{i}-c_{j}∥}^{-\frac{2}{m-1}}}{\sum_{k=1}^{C}{∥x_{i}-c_{k}∥}^{-\frac{2}{m-1}}}\\ c_{j}=\frac{\sum_{i=1}^{L}w_{{}_{ij}}^{m}x_{i}}{\sum_{i=1}^{L}w_{{}_{ij}}^{m}} \end{array}\right..$$

### 2.2. Kernel K-Means and Kernel Fuzzy C-Means

To improve performance of k-means and fuzzy c-means in linearly inseparable datasets, we may develop their kernel versions by choosing a feature mapping $\varphi \left(\cdot \right):R^{D}\to H$ from data points to kernel Hilbert space [37]. Though φ is usually unknown, it must satisfy

(5) $$K\left(x,y\right)={\left(\varphi (x)\right)}^{T}\varphi \left(y\right)$$

where K(x,y) is a kernel function. Commonly used kernel functions are presented in Table 1.

In Table 1, $\langle x,y\rangle =x\cdot y$ denotes the inner product of x and y, and σ,α,β are parameters of the kernel. The objective function of kernel k-means is defined as

(6) $$J_{k}=\sum_{j=1}^{K}\sum_{x_{i}\in \omega_{j}}{∥\varphi (x_{i})-c_{j}∥}^{2}$$

where

(7) $$c_{j}=\frac{1}{L_{j}}\sum_{x_{i}\in \omega_{j}}\varphi (x_{i}).$$

Using (5) and (7), we have

(8) $$\begin{array}{c} {∥\varphi (x_{i})-c_{j}∥}^{2}=K\left(x_{i},x_{i}\right)-\frac{2}{L_{j}}\sum_{x_{k}\in \omega_{j}}^{L}K(x_{k},x_{i})+\frac{1}{{\left(L_{j}\right)}^{2}}\sum_{x_{l}\in \omega_{j}}^{L}\sum_{x_{h}\in \omega_{j}}^{L}K(x_{l},x_{h}) \end{array}.$$

Let $w_{ij}$ represent the membership degree of the *i*-th point belonging to the *j*-th class. Likewise, we can define the kernel FCM clustering model as follows.

(9) $$\begin{array}{c} \min\;J_{f}\left(W\right)=\sum_{j=1}^{C}\sum_{i=1}^{L}w_{ij}^{m}{∥\varphi (x_{i})-c_{j}∥}^{2}\\ s.t.\sum_{j=1}^{C}w_{ij}=1,w_{ij}\geq 0,m>1 \end{array}$$

where

(10) $$c_{j}=\frac{\sum_{l=1}^{L}w_{lj}^{m}\varphi (x_{l})}{\sum_{l=1}^{L}w_{lj}^{m}},$$

(11) $$\begin{array}{c} {∥\varphi (x_{i})-c_{j}∥}^{2}=K\left(x_{i},x_{i}\right)-\frac{2}{\sum_{l=1}^{L}w_{lj}^{m}}\sum_{k=1}^{L}w_{kj}^{m}K\left(x_{k},x_{i}\right)+\frac{1}{{\left(\sum_{l=1}^{L}w_{lj}^{m}\right)}^{2}}\sum_{l=1}^{L}\sum_{h=1}^{L}w_{lj}^{m}w_{hj}^{m}K\left(x_{l},x_{h}\right) \end{array}.$$

The membership degree is computed via

(12) $$w_{ij}=\frac{{\left({∥\varphi \left(x_{i}\right)-c_{j}∥}^{2}\right)}^{-\frac{1}{m-1}}}{\sum_{k=1}^{C}{\left({∥\varphi \left(x_{i}\right)-c_{k}∥}^{2}\right)}^{-\frac{1}{m-1}}}.$$

## 3. Kernel Probabilistic K-Means

In this section, the kernel probabilistic k-means (KPKM) are proposed.

We first review the problem. When m=1, the KFCM model gets into a special case, namely,

(13) $$\begin{array}{c} \min\;J_{f}\left(W\right)=\sum_{j=1}^{K}\sum_{i=1}^{L}w_{ij}{∥\varphi (x_{i})-c_{j}∥}^{2}\\ s.t.\sum_{j=1}^{K}w_{ij}=1,w_{ij}\geq 0 \end{array}$$

where

(14) $$c_{j}=\frac{\sum_{l=1}^{L}w_{lj}\varphi \left(x_{l}\right)}{\sum_{l=1}^{L}w_{lj}},$$

(15) $$\begin{array}{c} {∥\varphi (x_{i})-c_{j}∥}^{2}=K\left(x_{i},x_{i}\right)-\frac{2}{\sum_{l=1}^{L}w_{lj}}\sum_{k=1}^{L}w_{kj}K\left(x_{k},x_{i}\right)+\frac{1}{{\left(\sum_{l=1}^{L}w_{lj}\right)}^{2}}\sum_{l=1}^{L}\sum_{h=1}^{L}w_{lj}w_{hj}K\left(x_{l},x_{h}\right) \end{array}.$$

This special case cannot be solved by Lagrangian optimization for m>1, because (12) cannot be computed when m=1 (when m=1, $\frac{1}{m-1}=\frac{1}{0}$ cannot be computed).

In this paper, we use the optimization methods to solve this problem, but the partial derivative of (13) with respect to $w_{ij}$ is ${∥\varphi (x_{i})-c_{j}∥}^{2}$, and it does not contain $w_{ij}$.

In order to solve the problem, we introduce (14) into (13), and redefine the member degrees $w_{ij}$ as probability $p_{ij}$ for 1≤i≤L and 1≤j≤K. Finally, we have

(16) $$\begin{array}{c} J\left(P\right)=\sum_{j=1}^{K}\sum_{i=1}^{L}p_{ij}{∥\varphi \left(x_{i}\right)-\frac{\sum_{l=1}^{L}p_{lj}\varphi \left(x_{l}\right)}{\sum_{l=1}^{L}p_{lj}}∥}^{2}\\ s.t.\;\sum_{j=1}^{K}p_{ij}=1,p_{ij}\geq 0 \end{array}$$

where probability vector

$$P={\left\{p_{11},\ldots ,p_{1K},p_{21},\ldots p_{2K},\ldots ,p_{LK}\right\}}^{T}.$$

(16) is the proposed kernel probabilistic k-means.

The proposed KPKM is a soft clustering method. The $p_{ij}\left(0\leq p_{ij}\leq 1\right)$ is the probability of the *i*-th data point belonging to the *j*-th cluster. The higher $p_{ij}$ is, the greater possibility of the *i*-th data point belonging to the *j*-th cluster. In KKM, the membership degree has only two values (0 and 1). In the proposed KPKM, $w_{ij}\in \left[0,1\right]$ (although final $w_{ij}=0\mathrm{or}1$).

## 4. A Fast Solution to KPKM

KPKM is a nonlinear programming problem with linear equalities and linear inequalities constraints, and it is able to be solved by active gradient projection [35,36] theoretically. In this section, on the basis of AGP, we first calculate the gradient of of the objection function of KPKM, and then design a fast AGP algorithm to solve the KPKM model.

The fast AGP has two advantages: iteratively updating the projection matrix and estimating the maximum step length.

### 4.1. Gradient Calculation

For convenience, we define $F_{kj}$ as

(17) $$F_{kj}={∥\varphi \left(x_{k}\right)-\frac{\sum_{l=1}^{L}p_{lj}\varphi \left(x_{l}\right)}{\sum_{l=1}^{L}p_{lj}}∥}^{2}.$$

Using the chain rule on (16), we obtain

(18) $$\frac{\partial J}{\partial p_{ij}}=\sum_{k=1}^{L}p_{kj}\frac{\partial F_{kj}}{\partial p_{ij}}+F_{ij}.$$

According to (17), we can further derive

(19) $$\begin{array}{c} \frac{\partial F_{kj}}{\partial p_{ij}}=\frac{-2}{\sum_{i=1}^{L}p_{ij}}\left(K\left(x_{k},x_{i}\right)-\frac{\sum_{l=1}^{L}p_{lj}K\left(x_{k},x_{l}\right)}{\sum_{i=1}^{L}p_{ij}}\right.\left.-\frac{\sum_{l=1}^{L}p_{lj}K\left(x_{i},x_{l}\right)}{\sum_{i=1}^{L}p_{ij}}+\frac{\sum_{l=1}^{L}\sum_{h=1}^{L}p_{lj}p_{hj}K\left(x_{l},x_{h}\right)}{{\left(\sum_{i=1}^{L}p_{ij}\right)}^{2}}\right) \end{array}.$$

By substituting (19) into (18), we finally get

(20) $$\begin{array}{c} \frac{\partial J}{\partial p_{i\;j}}\;=\;K\;\left(x_{i},x_{i}\right)\;+\;\frac{1}{{\left(\sum_{l=1}^{L}p_{l\;j}\right)}^{2}}\;\sum_{l=1}^{L}\;\sum_{h=1}^{L}p_{l\;j}p_{h\;j}K\left(x_{l},x_{h}\right)-\frac{2}{\sum_{l=1}^{L}p_{lj}}\sum_{k=1}^{L}p_{kj}K\left(x_{k},x_{i}\right) \end{array}.$$

and the gradient

(21) $$\nabla J={\left[\begin{array}{ccccccc} \frac{\partial J}{\partial p_{11}} & \cdots & \frac{\partial J}{\partial p_{1K}} & \cdots & \frac{\partial J}{\partial p_{Lj}} & \cdots & \frac{\partial J}{\partial p_{LK}} \end{array}\right]}^{T}.$$

### 4.2. Fast AGP

In the constraints of KPKM, there are *L* linear equalities and *K×L* linear inequalities,

(22) $$\forall 1\leq i\leq L,\sum_{j=1}^{K}p_{ij}=1,$$

(23) $$\forall 1\leq i\leq L,1\leq j\leq K,p_{ij}\geq 0.$$

Let ϕ=K×L. Let $I_{\phi \times \phi }$ be the identity matrix of size ϕ×ϕ. Define two matrices, inequality matrix A and equality matrix E, where

(24) $$A=I_{\phi \times \phi },$$

(25) $$E={\left[\begin{array}{ccc} \underset{K}{\underbrace{\begin{array}{ccc} 1 & \ldots & 1 \end{array}}} & \begin{array}{ccc} 0 & \ldots & 0 \end{array} & \begin{array}{ccc} 0 & \ldots & 0 \end{array}\\ \underset{K}{\underbrace{\begin{array}{ccc} 0 & \ldots & 0 \end{array}}} & \underset{K}{\underbrace{\begin{array}{ccc} 1 & \ldots & 1 \end{array}}} & \begin{array}{ccc} 0 & \ldots & 0 \end{array}\\ & \ldots & \\ \begin{array}{ccc} 0 & \ldots & 0 \end{array} & \begin{array}{ccc} 0 & \ldots & 0 \end{array} & \underset{K}{\underbrace{\begin{array}{ccc} 1 & \ldots & 1 \end{array}}} \end{array}\right]}_{L\times \phi }.$$

Note that each row of A corresponds to one and only one inequality in (23), and each row of E corresponds to one and only one equality in (22). Accordingly, the KPKM’s constraints can be simply expressed as

(26) AP≥0,EP=1

where $1={\left[1,\ldots ,1\right]}_{L\times 1}^{T}$. Let $P^{\left(0\right)}$ stand for a randomly initialized probability vector, and $P^{\left(k\right)}$ for the probability vector at iteration *k*. The rows of inequality matrix A can be broken into two groups: one is active; the other is inactive. The active group is composed of all inequalities that must work exactly as an equality at $P^{\left(k\right)}$, whereas the inactive group is composed of the left inequalities. If $A_{1}^{\left(k\right)}$ and $A_{2}^{\left(k\right)}$ respectively denote the active group and the inactive group, we have

(27) $$A_{1}^{\left(k\right)}P^{\left(k\right)}=0,$$

(28) $$A_{2}^{\left(k\right)}P^{\left(k\right)}>0.$$

At iteration *k*, the active matrix $N^{\left(k\right)}$ is defined as

(29) $$N^{\left(k\right)}=\left[\begin{array}{c} A_{1}^{\left(k\right)}\\ E \end{array}\right].$$

When $N=N^{\left(k\right)}$ is not a square matrix, we can construct its projection matrix $G^{\left(k\right)}$ and the corresponding orthogonal projection matrix $Q^{\left(k\right)}$ as follows:

(30) $$G^{\left(k\right)}=N^{T}{\left(NN^{T}\right)}^{-1}N,$$

(31) $$Q^{\left(k\right)}=I-G^{\left(k\right)}.$$

Suppose that n from $A_{2}^{\left(k-1\right)}$ is the active row vector at iteration k, satisfying $n^{T}P^{\left(k-1\right)}>0$ and $n^{T}P^{\left(k\right)}=0$. According to matrix theory [38], we can more efficiently compute $G^{\left(k\right)}$ and $Q^{\left(k\right)}$ by

(32) $$\begin{array}{c} G^{\left(k\right)}=G^{\left(k-1\right)}+Q^{\left(k-1\right)}n^{T}{\langle Q^{\left(k-1\right)}n^{T},Q^{\left(k-1\right)}n^{T}\rangle }^{-1}nQ^{\left(k-1\right)} \end{array}.$$

Furthermore, we can compute the projected gradient by

(33) $$d^{\left(k\right)}=-Q^{\left(k\right)}\nabla J\left(P^{\left(k\right)}\right).$$

Using $d^{\left(k\right)}$, we update the probability vector $P^{\left(k+1\right)}$ as

(34) $$P^{\left(k+1\right)}=P^{\left(k\right)}+t^{\left(k\right)}d^{\left(k\right)}$$

where $t^{\left(k\right)}$ is the step length. Usually, $t^{\left(k\right)}$ is chosen as a small number. For fast convergence, we estimate the maximum step length as follows.

(35) $$t^{\left(k\right)}=t_{\max}^{\left(k\right)}.$$

(1) Let $p_{ij}^{\left(k+1\right)}=p_{ij}^{\left(k\right)}+t_{ij}d_{ij}^{\left(k\right)}=0$ for $p_{ij}^{\left(k\right)}>0$;
(2) Compute tij=−pij(k)pij(k)dij(k)dij(k) for $p_{ij}^{\left(k\right)}>0$ and $d_{ij}^{\left(k\right)}<0$;
(3) $t_{\max}^{\left(k\right)}=\min\left\{t_{ij}\right\}$.

**Figure 1:**
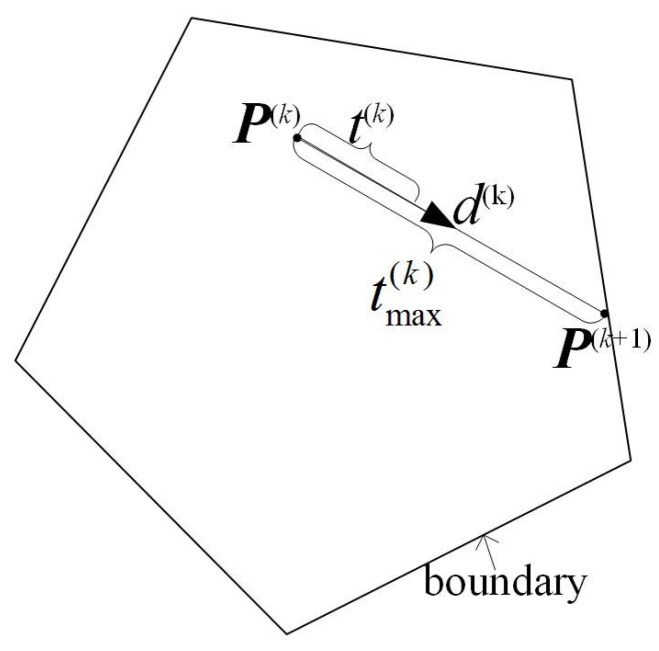
Maximum step length and small step length.

As shown in Figure 1, maximum step length is compared with small step length.

When $N=N^{\left(k\right)}$ is a square matrix, it must be invertible. In this case, we actually have $G^{\left(k\right)}=N^{T}{\left(NN^{T}\right)}^{-1}N=I_{LK\times LK}$, and $Q^{\left(k\right)}=0$. Thus, $d^{\left(k\right)}=-Q^{\left(k\right)}\nabla J\left(P^{\left(k\right)}\right)$=0 is not a feasible descent direction. A new descent direction can be computed as follows:

(1) Compute a new vector,
  (36) $$q^{\left(k\right)}={\left(NN^{T}\right)}^{-1}N\nabla J={\left(N^{T}\right)}^{-1}\nabla J.$$
(2) Break $q^{\left(k\right)}$ into two parts $q_{1}^{\left(k\right)}$ and $q_{2}^{\left(k\right)}$, namely,
  (37) $$q^{\left(k\right)}={\left[\begin{array}{cc} {\left(q_{1}^{\left(k\right)}\right)}^{T} & {\left(q_{2}^{\left(k\right)}\right)}^{T} \end{array}\right]}^{T}$$
  where the size of $q_{1}^{\left(k\right)}$ is the number of rows of $A_{1}^{\left(k\right)}$, and that of $q_{2}^{\left(k\right)}$ is the number of rows of E.
(3) If $q_{1}^{\left(k\right)}\geq 0$, stop. Otherwise, choose any element from $q_{1}^{\left(k\right)}$ that is less than 0 and delete the corresponding row of $A_{1}^{\left(k\right)}$; then use (29)–(31) and (33) to compute $d^{\left(k\right)}$.

The above fast AGP solution to KPKM is outlined in Algorithm 1. Compared with the original AGP, the fast AGP has two advantages: iteratively updating the projection matrix (shown in (32)) and estimating the maximum step length (shown in (35)).

### 4.3. Analysis of Complexity

In this section, the computational complexities for the traditional AGP algorithm and the proposed FAGP algorithm are analyzed. Let $T_{A}$ represent the iteration number of AGP. Let $T_{F}$ represent the iteration number of FAGP. In computations of all matrices, the computational complexity of the projection matrix G is the highest. In the AGP algorithm, computing G via (30) requires $O(K^{3}L^{3})$, so the total computational complexity for AGP algorithm is $O(T_{A}K^{3}L^{3})$. In the FAGP algorithm, G is computed via (32), and it does not require to compute the inverse of matrices (i.e., ${\left({\mathrm{NN}}^{T}\right)}^{-1}$), and computing G via (32) requires $O(K^{3}L^{2})$, so the total computational complexity for FAGP algorithm (i.e., Algorithm 1) is $O(T_{F}K^{3}L^{2})$.
**Algorithm 1** Fast active gradient projection (FAGP).      **Input:**
***X*** and ***K***      **Do:**            (1) Let k=0, $N=N^{\left(0\right)}=E$,$G^{\left(0\right)}=N^{T}{\left(NN^{T}\right)}^{-1}N$,      initialize $P^{\left(0\right)}$, go to (4);            (2) Find $n\in A_{2}^{\left(k-1\right)}$ meeting $n^{T}P^{\left(k-1\right)}>0$ and $n^{T}P^{\left(k\right)}=0$;            (3) Compute $G^{\left(k\right)}$ by (32);
            (4) Compute $Q^{\left(k\right)}$ by (31), and $d^{\left(k\right)}$ by (33);
            (5) Compute $t^{\left(k\right)}$ by (35), and $P^{\left(k+1\right)}$ by (34);
            (6) Let k=k+1, if $G^{\left(k\right)}\neq I_{LK\times LK}$, go to (2);            (7) Construct $N=N^{\left(k\right)}$ by (29), and by (36);            (8) Break into $q_{1}^{\left(k\right)}$ and $q_{2}^{\left(k\right)}$ by (37);            (9) If all elements of $q_{1}^{\left(k\right)}$ are more than 0, stop;            (10) Choose any element less than 0 from $q_{1}^{\left(k\right)}$,      and delete the corresponding row of $A_{1}^{\left(k\right)}$;            (11) Reconstruct $N=N^{\left(k\right)}$ by (29);            (12) Compute $G^{\left(k\right)}$ by (30), and $Q^{\left(k\right)}$ by (31);            (13) Compute $d^{\left(k\right)}$ by (33), and $t^{\left(k\right)}$ by (35);            (14) Compute $P^{\left(k+1\right)}$ by (34);            (15) Let k=k+1, and go to (7).      **Output:** the probability vector P


## 5. Experimental Results

In order to evaluate performance of the proposed KPKM model (equivalent to KFCM at *m* = 1) solved by the FAGP algorithm, we have conducted a lot of experiments on one synthetic dataset, ten UCI datasets (http://archive.ics.uci.edu/ml (accessed on 8 March 2021)) and the MNIST dataset (http://yann.lecun.com/exdb/mnist/ (accessed on 8 March 2021)). These datasets are detailed in Section 5.1, Section 5.2 and Section 5.3. In Section 5.1, we use a synthetic dataset to compare KPKM using a Gaussian kernel with KPKM using a linear kernel. In Section 5.2 and Section 5.3, we compare the proposed KPKM with KFCM, KKM, FCM, and KM to evaluate the performance of KPKM solved by the proposed fast AGP. Moreover, the Section 5.4 and Section 5.5 evaluate the descent stability and convergence speed of the proposed FAGP, respectively. In the experiments, we implemented our own MATLAB code for KPKM, KFCM, and KKM with two build-in functions, *sparse* and *full*, employed for matrix optimization. Moreover, we called MATLAB’s build-in functions *fcm* and *kmeans* for FCM and KM, respectively.

All experiments were carried out on a PC with an Intel(R) Core(TM) i7-4790 CPU at 3.60 GHz, 8.00 G RAM, running Windows 7 and MATLAB 2015a.

### 5.1. The Experiment on the Synthetic Dataset

**Figure 2:**
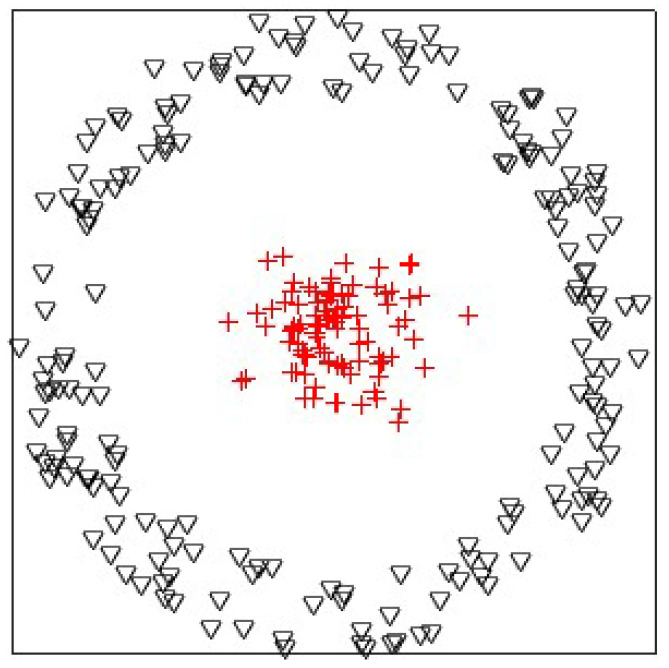
The synthetic dataset, which is composed of two linearly inseparable classes: disc and ring.

**Figure 3:**
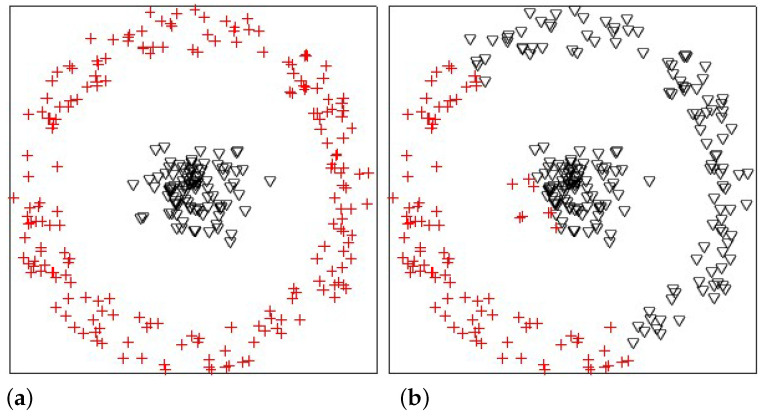
The results of the synthetic dataset clustered by Gaussian radial basis function kernel probabilistic k-means (KPKM) (**a**) and linear KPKM (**b**).

In this experiment, we analyzed the influences of the Gaussian radial basis function kernel and the linear kernel on KPKM when clustering one synthetic dataset, which is shown in Figure 2. The synthetic dataset contained 300 points, with 100 and 200 being from two linearly inseparable classes, disc and ring, respectively. The result is displayed in Figure 3. Obviously, Gaussian radial basis function KPKM can cluster perfectly, whereas linear KPKM cannot.

### 5.2. Experiment on Ten UCI Datasets

In this experiment, we compare the clustering performance of KPKM with the performances of KFCM, KKM, FCM, and KM in terms of three measures: normalized mutual information (NMI), adjusted Rand index (ARI), and v-measure (VM) [39,40]. NMI is a normalization of the mutual information score to scale the results between 0 (no mutual information) and 1 (perfect correlation). The NMI is defined as

(38) $$\mathrm{NMI}\left(U,V\right)=\frac{\mathrm{MI}(U,V)}{\mathrm{mean}(H(U),H(V))}$$

where H(U) is the entropy of *U*; MI is given by

(39) $$\mathrm{MI}\left(U,V\right)=\sum_{i=1}^{\left|U\right|}\sum_{j=1}^{\left|V\right|}\frac{|U_{i}\cap V_{j}|}{N}\log\frac{N|U_{i}\cap V_{j}|}{|U_{i}\left|\right|V_{j}|}$$

where $|U_{i}|$ is the number of the samples in cluster $U_{i}$. ARI is an adjusted similarity measure between two clusters. The ARI is defined as

(40) $$\mathrm{ARI}=\frac{\mathrm{RI}-E(\mathrm{RI})}{\max\left(\mathrm{RI}\right)-E\left(\mathrm{RI}\right)}$$

where RI is the ratio of data points clustered correctly to all data points, and *E*(RI) is the expectation of RI. VM is a harmonic mean between homogeneity and completeness, where homogeneity means that each cluster is a subset of a single class, and completeness means that each class is a subset of a single cluster. The VM is defined as

(41) $$\mathrm{VM}=\frac{(1+\beta )\times \mathrm{homogeneity}\times \mathrm{completeness}}{\left(\beta \times \mathrm{homogeneity}+\mathrm{completeness}\right)}$$

The higher the NMI, ARI and VM, the better the performance.

We describe the ten UCI datasets represented in Table 2 by name, code, number of instances, number of classes and number of dimensions. A set of experiential parameters was selected. We set *m* = 2 for KFCM, and *m* = 1.3 for FCM. With the Gaussian radial basis function kernel, we also selected appropriate σ (shown in Table 3), and report the clustering results in Table 4. The better results in each case is highlighted in bold. (The performance of many a method depends on parameters [41]. By experiments we found that for different algorithms with the same kernel function, the most appropriate parameters are usually different, so we used different settings to make the methods have the best clustering performances they could. We also tried to select the most appropriate parameter by using our experience.) We ran every algorithm 10 times, and average results were calculated.

From Table 4, we can see that

(1) For NMI, KPKM had the best clustering results on nine datasets, so the clustering performance of KPKM was the best for NMI.
(2) For ARI, KPKM had the best clustering results on six datasets. KFCM, KKM, FCM, and KM performed the best on 2, 1, 0, and 1 datasets, respectively, so KPKM performed the best for ARI.
(3) For VM, KPKM, KFCM, KKM, FCM, and KM had the best clustering results on 7, 0, 2, 1, and 0 datasets, respectively. Thus, KPKM had the best clustering performance for VM.

Overall, KPKM is better than the other models.

### 5.3. Experiment on the MNIST Dataset

**Figure 4:**
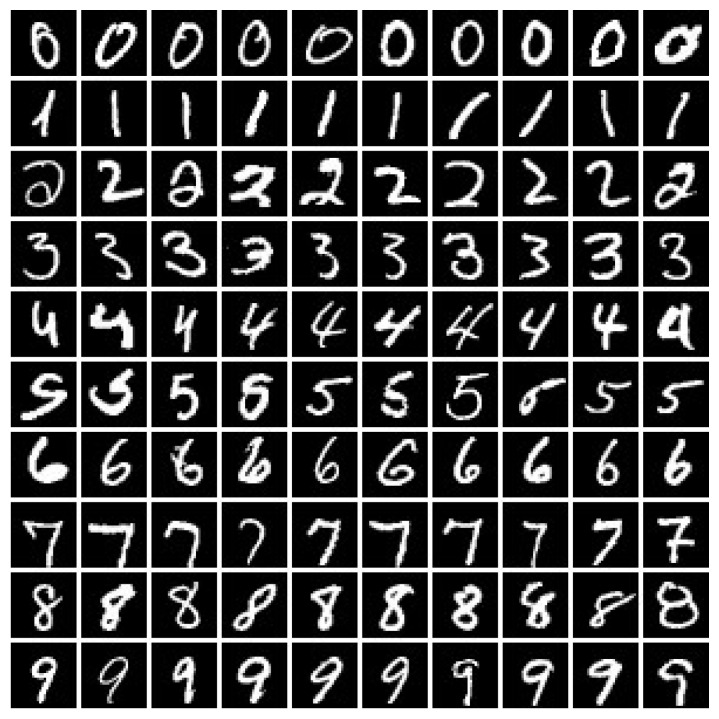
One-hundred digital examples, 10 per class.

In this experiment, we used the MNIST dataset to compare KPKM with KFCM and KKM when clustering digital images. The MNIST dataset was composed of handwritten digits, with 60,000 examples for training and 10,000 examples for testing. All digits were size-normalized and centered in fixed-size images. To evaluate the clustering performances of KPKM, KFCM, and KKM, we randomly chose 1000 training examples; 100 are shown in Figure 4.

**Figure 5:**
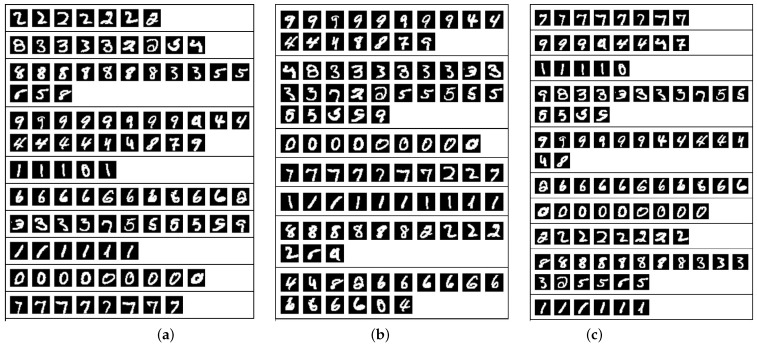
The results of the examples of Figure 2 clustered by KPKM (**a**), KFCM (**b**), and KKM (**c**).

Moreover, we defined a CWSS kernel based on complex wavelet structural similarity (CWSS) [42]. CWSS may be regarded as a coefficient that does not change the structural content of an image. By using CWSS(x,y) to denote the similarity between two images x and y, we can express the CWSS kernel as

(42) $$K_{\mathrm{cwss}}\left(x,y\right)=\exp\left(-\frac{1-CWSS(x,y)}{2\sigma^{2}}\right)$$

where σ=5 is set for KFCM, and σ=1 for both KPKM and KKM. Additionally, *m* = 1.3 is set for KFCM. We present the results in Table 5, with the examples of Figure 4 correspondingly being displayed in Figure 5. From Table 5, we can see that KPKM outperformed KFCM and KKM in terms of NMI, ARI, and VM. From Figure 5, we can observe that both KPKM and KKM found ten clusters, whereas KFCM found only seven clusters, although the number of clusters was set to ten.

### 5.4. Experiment for Descent Stability

**Figure 6:**
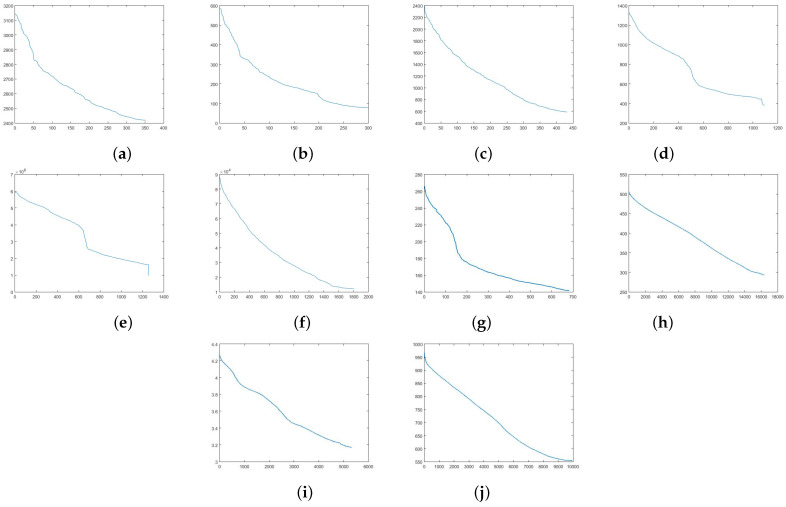
Descent stability of FAGP on 6 UCI datasets: Ionosphere (**a**), Iris (**b**), Seeds (**c**), Glass (**d**), Segmentation (**e**), Dermatology (**f**), Breast (**g**), Natural (**h**), Yeast (**i**), and Waveform (**j**). The x-axis shows the iteration number. The y-axis shows the objective function value.

In this experiment, we used 10 UCI datasets to evaluate descent stability of FAGP. AGP selects a small step length to converge. If AGP selects a large step length, the objective function value may descend with oscillation, and even does not converge. FAGP iteratively estimates a maximum step length at each iteration for speeding up its convergence. However, does it have any serious influence on the convergence? we ran the proposed FAGP on 10 UCI datasets to demonstrate the descent stability of FAGP. As shown in Figure 6, we can see that FAGP descends stably without oscillation at each iteration.

### 5.5. Convergence Speed Comparison between FAGP and AGP

In this experiment, we compared the convergence speeds of FAGP and AGP by using running time on 10 UCI datasets. η is the ratio of FAGP’s running time to AGP’s. FAGP and AGP used the same initializations in each case. The results are presented in Table 6 and Table 7 (“–” means the running time was too long to obtain the final clustering results), and we can observe the proposed FAGP ran faster than AGP on all the 6 datasets. For Iris and Seeds datasets, FAGP used less than 10% of running time of AGP. FAGP also required fewer iterations than AGP. For large datasets, the running time of AGP was too long, but the proposed fast AGP could obtain the final clustering results.

## 6. Conclusions

In this paper, a novel clustering model (i.e., KPKM) was proposed. The proposed KPKM solves the problem of KFCM for *m* = 1, and this problem cannot be solved by existing method. The traditional AGP method can solve the proposed KPKM, but the efficiency of AGP is low. A fast AGP was proposed to speed up the AGP. The proposed fast AGP uses a maximum step length strategy to reduce the iteration number and uses an iterative method to update the projection matrix. The experimental results demonstrated that the fast AGP is able to solve the KPKM and the fast AGP requires less running time than AGP (the proposed FAGP requires 4.22–27.90% of the running time of AGP on real UCI datasets). The convergence of the proposed method was also analyzed by experiments. Additionally, in the experiments, the KPKM model could produce overall better clustering results than the other models, including KFCM, KKM, FCM, and KM. The proposed KPKM obtained the best clustering results on at least 6 real UCI datasets (a total of 10 real UCI datasets were used).

As future work, the proposed KPKM using other kernels will be evaluated in a variety of applications. For large datasets, the proposed method still has some disadvantages, so the next project will include speeding up the AGP on large datasets.

## Figures and Tables

**Table 1:** Commonly used kernel functions.

Name	Code
Linear kernel	$K\left(x,y\right)=\langle x,y\rangle$
Laplace Radial Basis Function kernel	$K_{\mathrm{lap}}\left(x,y\right)=\exp\left(-\sigma ∥x-y∥\right)$
Gaussian Radial Basis Function kernel	$K_{\mathrm{gau}}\left(x,y\right)=\exp\left(-\frac{{∥x-y∥}^{2}}{2\sigma^{2}}\right)$
Polynomial kernel	$K_{\mathrm{pol}}\left(x,y\right)={\left(x\cdot y+\beta \right)}^{\alpha }$
Sigmoid kernel	$K_{\tan}\left(x,y\right)=\tanh\left(\alpha x\cdot y+\beta \right)$

**Table 2:** The ten UCI datasets used.

Name	Code	Instances	Classes	Dimensions
Iris	R1	150	3	4
Seeds	R2	210	3	7
Segmentation	R3	210	7	19
Glass	R4	214	6	9
Ionosphere	R5	351	2	33
Dermatology	R6	358	6	34
Breast-cancer	R7	683	2	9
Natural	R8	2000	9	294
Yeast	R9	2426	3	24
Waveform	R10	5000	3	21

**Table 3:** Parameter σ selected for kernel clustering methods.

Dataset	KPKM	KFCM	KKM
R1	1.08	1.22	0.9
R2	1.9	2	2
R3	510	530	540
R4	510	100	510
R5	1.5	1	1.3
R6	3.3	2	18
R7	12	10	15
R8	3	0.9	2.7
R9	15	10	25
R10	10	15	13

**Table 4:** Comparisons of KPKM with kernel fuzzy c-means (KFCM), kernel k-means (KKM), fuzzy c-means (FCM), and k-means (KM) on ten UCI datasets (DS) in terms of normalized mutual information (NMI), adjusted Rand index (ARI), and v-measure (VM).

DS	Method	KPKM	KFCM	KKM	FCM	KM
R1	NMI	**0.8146**	0.7900	0.7820	0.6723	0.6733
	ARI	**0.8119**	0.8015	0.7590	0.5763	0.5779
	VM	**0.8146**	0.7900	0.7820	0.7081	0.7149
R2	NMI	**0.7073**	0.6949	0.7038	0.6949	0.7025
	ARI	0.7141	0.7166	**0.7231**	0.7166	0.7135
	VM	**0.7073**	0.6949	0.7038	0.6949	0.6999
R3	NMI	**0.5555**	0.5503	0.5154	0.4678	0.5132
	ARI	0.3909	**0.4141**	0.3429	0.3172	0.3313
	VM	0.5553	0.5503	0.5139	**0.5729**	0.5252
R4	NMI	**0.4436**	0.3594	0.3943	0.3489	0.4178
	ARI	**0.2796**	0.2137	0.2542	0.2126	0.2551
	VM	**0.4424**	0.3593	0.3934	0.3807	0.3857
R5	NMI	0.2476	0.2390	**0.2715**	0.1299	0.1349
	ARI	0.1747	0.1098	0.1657	0.1727	**0.1777**
	VM	0.2476	0.2390	**0.2715**	0.1298	0.1348
R6	NMI	**0.2919**	0.2068	0.2778	0.1046	0.1032
	ARI	**0.1795**	0.1388	0.1698	0.0261	0.0266
	VM	**0.2919**	0.2065	0.2776	0.1095	0.1056
R7	NMI	**0.7903**	0.7825	0.7741	0.7478	0.7478
	ARI	**0.8796**	0.8741	0.8674	0.8464	0.8464
	VM	**0.7903**	0.7825	0.7741	0.7478	0.7478
R8	NMI	**0.0531**	0.0326	0.0556	0.0521	0.0536
	ARI	0.0253	**0.0303**	0.0283	0.0273	0.0261
	VM	0.0525	0.0312	**0.0550**	0.0495	0.0529
R9	NMI	**0.0052**	0.0031	0.0050	0.0043	0.0050
	ARI	**0.0118**	0.0084	0.0118	0.0109	0.0117
	VM	**0.0052**	0.0031	0.0050	0.0045	0.0045
R10	NMI	**0.3654**	0.3162	0.3637	0.3606	0.3622
	ARI	**0.2546**	0.2377	0.2541	0.2529	0.2536
	VM	**0.3654**	0.3162	0.3637	0.3559	0.3622

**Table 5:** Comparisons of KPKM with KFCM and KKM on the MNIST dataset in terms of NMI, ARI, and VM.

Measure	KPKM	KFCM	KKM
NMI	**0.6830**	0.5438	0.6613
ARI	**0.5685**	0.4047	0.5256
VM	**0.6829**	0.5411	0.6613

**Table 6:** Running time (s) comparison of FAGP and AGP.

DS	FAGP	AGP	η
Ionosphere	0.719763	2.579700	27.90%
Iris	0.397728	9.410401	4.22%
Seeds	0.693613	8.650179	8.01%
Glass	3.482856	22.644511	15.38%
Segmentation	4.706316	19.125480	24.60%
Dermatology	10.659099	50.796225	20.98%
Breast	1.0153	6.1325	16.55 %
Natural	2309	–	–
Yeast	197.37	–	–
Waveform	213.64	–	–

**Table 7:** Comparison of the numbers of iterations used by FAGP and AGP.

DS	FAGP	AGP
Ionosphere	354	975
Iris	357	6506
Seeds	423	4061
Glass	1123	5616
Segmentation	1275	3986
Dermatology	1768	6784
Breast	682	694
Natural	16,378	–
Yeast	5609	–
Waveform	9939	–

## Data Availability

Not applicable.

## Abbreviations

The following abbreviations are used in this manuscript:

X	dataset
$x_{i}$	*i*-th data points of X
φ(·)	feature mapping from data points to kernel Hilbert space
$K_{\mathrm{lap}}\left(\cdot ,\cdot \right)$	Laplace Radial Basis Function kernel
$K_{\mathrm{gau}}\left(\cdot ,\cdot \right)$	Gaussian Radial Basis Function kernel
$K_{\mathrm{pol}}\left(\cdot ,\cdot \right)$	polynomial kernel
$K_{\tan}\left(\cdot ,\cdot \right)$	sigmoid kernel
$\omega_{j}$	*j*-th cluster
*L*	number of elements in X
$L_{j}$	number of elements in $\omega_{j}$
$c_{j}$	center of $\omega_{j}$
*K* and *C*	number of clustering center
W	membership degree matrix
$w_{ij}$	membership degree to the *i*-th data point in the *j*-th cluster
A	inequality matrix
E	equality matrix
P	probability matrix
I	identity matrix
$p_{ij}$	probability to the *i*-th data point in the *j*-th cluster
G	projection matrix
Q	orthogonal projection matrix
N	active matrix
N	row vector of N
d	projected gradient
*t*	step length
